# Expression of Epithelial and Mesenchymal Markers in Plasmatic Extracellular Vesicles as a Diagnostic Tool for Neoplastic Processes

**DOI:** 10.3390/ijms24043578

**Published:** 2023-02-10

**Authors:** Begoña O. Alen, Lara Sofía Estévez-Pérez, María Otero Alén, Saioa Domínguez Hormaetxe, Laureano Simón, Ángel Concha

**Affiliations:** 1 Molecular Biology Area, Department of Anatomical Pathology, Biomedical Research Institute A Coruña (INIBIC), University Hospital Complex A Coruña, 15006 A Coruña, Spain; 2 Santiago de Compostela Health Research Institute (IDIS), University Hospital Complex Santiago de Compostela, 15706 Santiago de Compostela, Spain; 3 Oncomatryx Biopharma, 48160 Derio, Spain

**Keywords:** extracellular vesicles, epithelial biomarker, mesenchymal biomarker, cancer diagnosis, liquid biopsy

## Abstract

Tumor-derived extracellular vesicles (TD-EVs) have active roles as cancer hallmark enablers. EVs RNA of epithelial and stromal cells carry information that facilitates the communication processes that contribute to oncological progression, so the objective of this work was to validate by RT-PCR the presence of epithelial (*KRT19*; *CEA*) and stromal (*COL1A2*; *COL11A1*) markers in RNA of plasmatic EVs in healthy and diverse-malignancy patients for the development of a non-invasive cancer diagnosis system using liquid biopsy. Ten asymptomatic controls and 20 cancer patients were included in the study, and results showed that the isolated plasmatic EVs by scanning transmission electron microscopy (STEM) andBiomedical Research Institute A Coruña nanoparticle tracking analysis (NTA) contained most exosome structures with also a considerable percentage of microvesicles. No differences were found in concentration and size distribution between the two cohorts of patients, but significant gene expression in epithelial and mesenchymal markers between healthy donors and patients with active oncological disease was shown. Results of quantitative RT-PCR are solid and reliable for *KRT19*, *COL1A2*, and *COL11A1*, so the analysis of RNA extracted from TD-EVs could be a correct approach to develop a diagnostic tool in oncological processes.

## 1. Introduction

Tissue biopsies have been the usual procedures when the onset of cancer symptoms appeared. However, these types of biopsies are invasive, expensive, and harmful, and sometimes they cannot even be performed due to the difficulty in accessing the tumor location or for patient health conditions [1]. In addition, due to the characteristics of neoplastic cells (heterogeneity, plasticity, etc.) and the different tumor genomic profiles, conventional biopsies are not capable of reflecting the complete nature of primary or secondary tumors, since they only represent a small part of the malignancy [2]. This fact makes difficult the correct patient management [3]. On the other hand, for monitoring response to therapy, rebiopsy becomes necessary to identify genetic alterations during and after treatment. Therefore, the development of new, lower-cost, non-invasive sampling techniques for screening, early diagnosis, investigation of tumor dynamics, and detection of the risk of recurrence was required [4]. Liquid biopsies emerged to overcome these drawbacks. This technique can be used as a method of screening/early detection [5], for monitoring responses to treatment, and detecting minimal residual disease or early recurrence, and it may offer molecular information at each moment of disease evolution [6,7]. Currently, most liquid biopsy studies in cancer are performed based on circulating tumor cells (CTCs), circulating tumor DNA (ctDNA), and extracellular vesicles (EVs) [8]. Between them, EVs offer particular advantages among CTCs or ctDNA. EVs are secreted by living cells, are found in large amounts in biofluids [9], and are more stable in circulating physiological conditions [8,10]. Furthermore, EVs, as well as CTCs or ctDNA, contain unique molecular cargo (nucleic acid, protein or lipids) from donor cells, playing a crucial role in mediating intercellular communication [11]. EVs are categorized into two classes: ectosomes and exosomes. Ectosomes (microvesicles (MVs) and oncosomes) are sized 100 to 1000 nm and originate from direct plasma membrane budding [12]. Exosomes are vesicles formed by endocytic pathways and range from 30 to 150 nm [12]. EVs have various functions depending on the cell of origin, and they have been intensively studied as facilitators of the immune response as well as their role in antigen presentation [11]. Their role in programmed cell death [13,14], angiogenesis [15], inflammation, and coagulation has also been reported [16,17]. Related to cancer, EVs facilitate tumorigenesis and tumor progression and are involved in the formation of premetastatic niches, tumor angiogenesis, and tumor immune suppression [18,19,20]. Tumor-derived extracellular vesicle (TD-EV) trafficking was observed in solid tumors [21]. They carry complex, bioactive cargo during tumor metastasis such as DNA, RNA, microRNA, and long non-coding RNA (lncRNA) [22] that allows crosstalk between malignant cells and can move to a distant metastatic lesion and modulate the tumor microenvironment (TME) to form a premetastatic niche. Thus, TD-EVs have roles as cancer hallmark enablers and may be potential targets for tumor development and metastasis, being studied as diagnostic, prognostic, or predictive cancer biomarkers [22].

EV RNA is a mixture of coding and noncoding RNA dependent of the microenvironment of donor cells [17]. It has also been reported that transported information via RNA facilitates the communication processes that contribute to oncological progression, being able to provide early diagnostic information as well as predictive outcomes [17]. In this sense, the study of neoplastic epithelial and stromal RNA markers in plasmatic TD-EVs could be used as a diagnostic tool in patients with active cancer disease. Epithelial biomolecules such as cytokeratin 19 (KRT19) and carcinoembryonic antigen (CEA) are involved in cancer progression [23]. KRT19 is one of the most used markers for the detection of dissemination of tumor cells in breast, lung, and colon cancer [24,25]. It is a type I keratin that is part of the cytoskeleton of numerous epithelial cells of different organs, and it is highly expressed in epithelial tumors, being a biomarker for detecting micro- and macro-metastasis in several malignancies [24]. Moreover, *KRT19* has been detected in exosomes derived from colon tumors by RT-PCR [26] and in circulating breast tumor cells [27]. CEA is a glycoprotein involved in cell adhesion [28] and is mainly expressed during fetal development. Plasma levels are very low in healthy adults [29]. It is considered a tumor biomarker in colorectal [23,29], pancreas [30], breast [31], and lung neoplasms [32], and was expressed in unfractionated plasma and EVs of patients with colon cancer [33].

The TME is formed by tumor cells, tumor stromal cells (stromal fibroblasts, endothelial cells, and immune cells), and the non-cellular components of the extracellular matrix (ECM) such as collagen, fibronectin, and laminin [34]. The non-malignant cells in the TME are known to promote tumorigenesis in all phases of cancer development and metastasis [35]. The ECM secreted by stromal cells plays a crucial role in tumor progression and metastasis as well as therapeutic resistance [36]. Type XI alpha 1 collagen (COL11A1) is a protein specifically expressed by fibroblasts associated with different types of malignant tumors (breast, pancreas, colon, lung, ovary, etc.), as evidenced by studies on tissues both at the RNA and protein levels [37,38]. Type I collagen (COL1A2) is a critical component of the ECM, and its expression is increased in pancreatic, colorectal, breast, and lung cancer [39,40,41,42].

In this context, the fact that TD-EVs can be used as diagnostic tumor markers, as can the presence of epithelial and stromal markers in RNA of plasmatic neoplastic EVs, the objective of this work was to validate by RT-PCR the presence of *KRT19*, *CEA*, *COL11A1*, and *COL1A2* in RNA of plasmatic EVs in healthy and diverse-malignancy patients for the development of a non-invasive cancer diagnosis system using liquid biopsy.

## 2. Results

### 2.1. Baseline Characteristics of Patients

Ten healthy donors as asymptomatic controls (AC) and 20 cancer patients (CP) were included in this study. All of them underwent blood extraction for healthcare purposes, and samples were analyzed in the Pathological Anatomy service at the University Hospital Complex of A Coruña. Clinical data such as gender, age, tumor histology, and disease stage were obtained from medical records and are summarized in Table 1.

Regarding the control group corresponding to healthy patients, ten patients aged between 25 and 55 years who had not previously presented any oncological process were analyzed. Patients also had no history of chronic pathologies, such as chronic inflammatory bowel disease (Crohn’s disease, ulcerative colitis, etc.), pancreatitis, cirrhosis, chronic obstructive pulmonary disease (COPD), or hypothyroidism, since it has been shown that these types of pathologies may be related to alterations in the basal levels of KRT19 [43,44] and CEA [45,46,47,48,49]. Among these 10 patients, 30% were men (*n* = 3) and 70% were women (*n* = 7).

In the group of cancer patients, we analyzed plasma samples of eight prostate adenocarcinomas (seven stage IV and one stage IIb); three renal cell carcinomas (two stage IV, one stage III); three pancreatic neuroendocrine tumors (stage IV); two melanomas (one stage IV and one in situ melanoma); one urothelial bladder carcinoma (stage IV); one patient with synchronous colorectal tumors, namely, one adenocarcinoma and one small intestine neuroendocrine tumor (stage I and stage IV, respectively); one serous ovarian carcinoma (stage III); and one small-cell lung carcinoma (stage IV). Among these 20 patients, 85.00% were men (*n* = 17) and 15.00% were women (*n* = 3). The staging of the different tumors was evaluated according to the eighth edition of the TNM classification of the American Joint Committee on Cancer of 2017 [50].

### 2.2. Characterization of Plasmatic Extracellular Vesicles

Visible and quantitative characterizations of plasmatic EVs were performed to assess purity and concentration. We isolated EVs from plasma of patients and STEM electron microscopy, and NTA were used to analyze the morphology, size distribution, and concentration of samples.

#### 2.2.1. Visible Characterization of Extracellular Vesicles by Electron Microscopy

**Figure 1:**
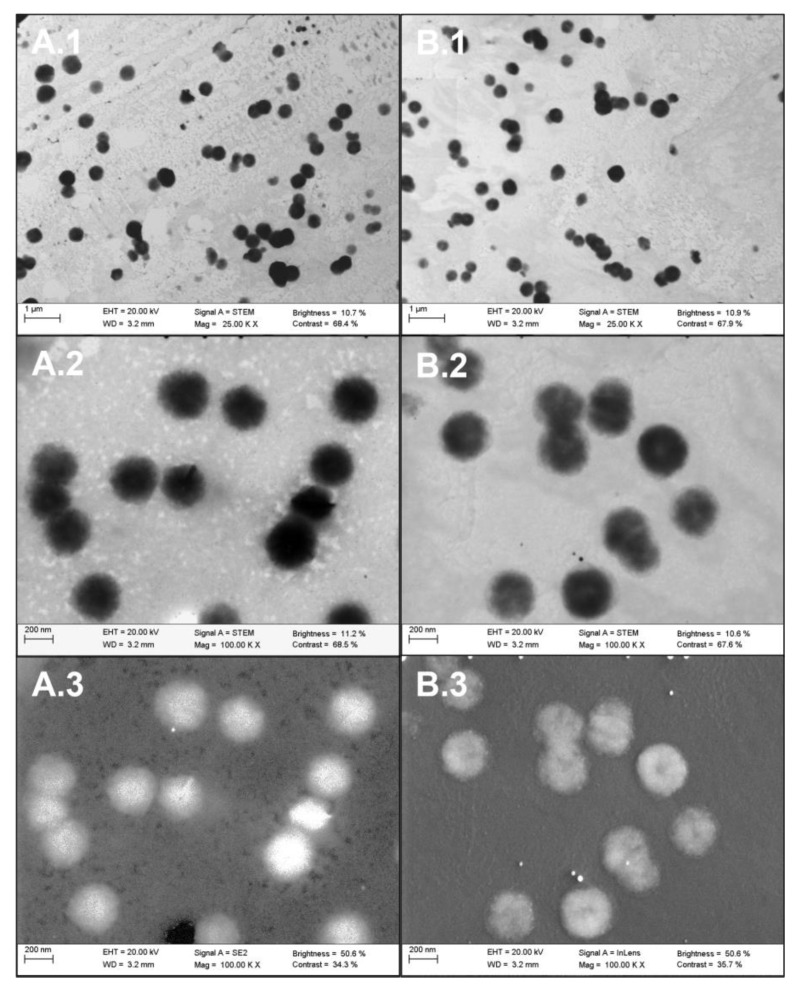
STEM micrographs of plasmatic EVs. A. EVs from asymptomatic controls (AC). (**A.1**) TEM micrography of mixed microvesicles and exosome structures at 25.00 K of magnification. Scale bar 1 μm. (**A.2**) TEM micrography of spheroid-shaped vesicles at 100.00 K of magnification. Scale bar 200 nm. (**A.3**) SEM micrography of spheroid-shaped vesicles at 100.00 K of magnification. Scale bar 200 nm. B. Plasmatic EVs from cancer patients (CP). (**B.1**) TEM micrography of mixed microvesicles and exosome structures at 25.00 K of magnification. Scale bar 1 μm. (**B.2**) TEM micrography of spheroid-shaped vesicles at 100.00 K of magnification. Scale bar 200 nm. (**B.3**) SEM micrography of spheroid-shaped vesicles at 100.00 K of magnification. Scale bar 200 nm. Images are representative for at least three independent experiments.

To confirm the presence, morphology, and size of EVs from plasma of cancer patients and healthy individuals, samples were observed by scanning transmission electron microscopy (STEM) technology. Vesicles ranging from 800 to 25 nm were analyzed. Micrographs showed the majority of exosome and microvesicle structures in both samples (Figure 1). The morphology revealed a greater number of spheroid EVs, with some cup-shaped exosome configurations (Figure 1A.1,A.2). No differences were observed in structure or morphology between AC and CP donors.

#### 2.2.2. Quantitative Characterization of Extracellular Vesicles by Nanoparticle Tracking Analysis (NTA)

**Figure 2:**
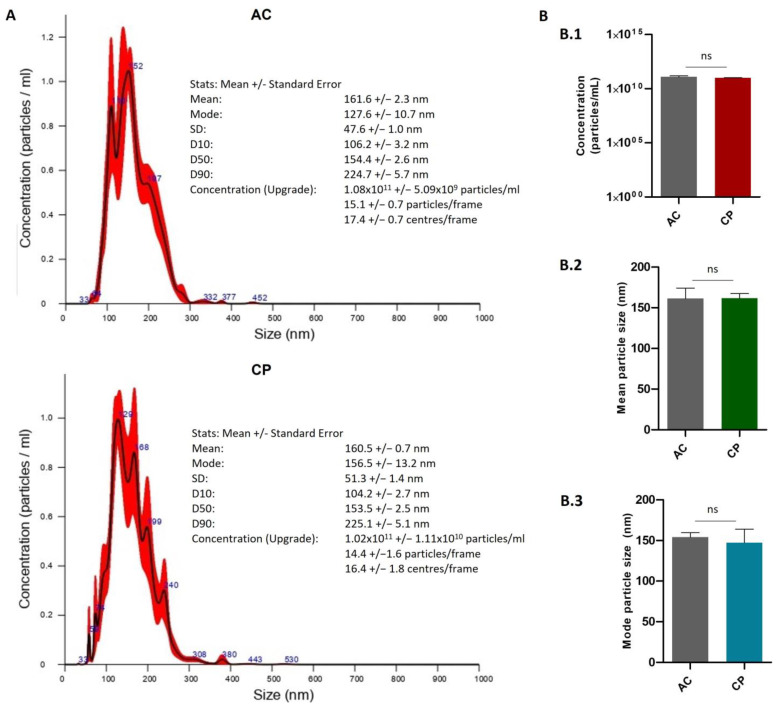
NTA of plasmatic EVs. (**A**) Concentration and size distribution of EVs in plasma of AC (**upper panel**) and CP (**lower pannel**) patients. (**B**) Differences in concentration (**B.1**) and size distribution (**B.2**,**B.3**) between AC and CP plasmatic samples. No differences were found between AC and CP plasmatic EV samples. The data were expressed as mean ± SD obtained from three independent experiments. Ns denotes no statistically significant differences when comparing CP with the control group.

Nanoparticle tracking analysis (NTA) is a particle tracking method for measuring the EV concentration and size distribution (Figure 2) [51]. From NTA analysis, it was shown that the mean size of vesicles ranged from 161.6 ± 2.3 nm (AC samples) to 160.5 ± 0.7 nm (CP) (Figure 2A). Mode values were similar (AC: 127.6 ± 10.7 nm; CP: 156.5 ± 13.2 nm). Analysis showed that exosome size structures were majority in both samples. Regarding concentration, AC samples showed a mean value of 1.08 × 10^11^ ± 5.09 × 10^9^ particles/mL. Plasma of cancer donors registered a mean of 1.02 × 10^11^ ± 1.11 × 10^10^ particles/mL. No statistically significant differences were found in terms of concentration and size distribution between the two cohorts of patients (Figure 2B).

### 2.3. RNA Analysis of Integrity

**Figure 3:**
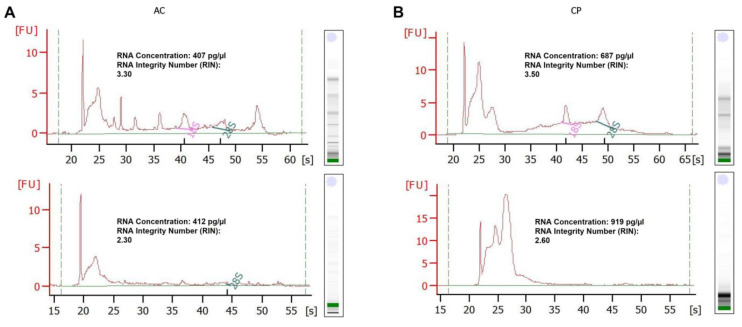
Bioanalyzer analysis of total RNA from plasmatic EVs by Agilent RNA Pico Chip. (**A**) Electropherograms of total EV RNA of two representative samples of AC controls. (**B**) Electropherograms for total EV RNA of two representative samples of CP donors. No differences were found in Bioanalyzer profiles between healthy and cancer patients.

In order to determine the integrity and concentration of total RNA extracted from plasmatic EVs, Bioanalyzer profiles were examined. RNA integrity number (RIN) values were analyzed for all plasma samples with RNA 6000 Pico Chip. Results showed that all samples seemed to be partially degraded with low RIN values, ranging from 2.3 to 4.2 (Figure 3). Plasmatic EVs harbored high amounts of small purified RNAs; this fact can have a negative impact on RIN values, since the algorithm behind RIN calculation assesses these small RNAs as degradation products [52]. Therefore, samples that appeared to be partially or total degraded actually showed a variable amount of small RNAs not reflected in the integrity analysis. In this context, the RIN value could not be the adequate method to quality control total EV RNA extracted samples. With respect to concentration, values ranging from 400 to 1.200 pg/µL were showed from the samples. Small concentrations of RNA were found, which was a drawback in terms of reproducibility and repeatability. No differences in integrity or concentration were found between samples from healthy donors and cancer patients (Figure 3A,B).

### 2.4. RNA Expression of Epithelial and Mesenchymal Markers

#### 2.4.1. RNA Expression Level in Plasma of AC vs. CP

The RNA relative expression of epithelial and mesenchymal biomarkers was calculated in plasma for healthy donors and cancer individuals by qRT-PCR. ΔCt values for AC and CP cohort are shown in Table 2 and Table 3, respectively. All data were normalized by *ACTB* relative gene expression.

ΔCt values were expressed as mean ± SEM. Healthy donors showed a ΔCt of 8.64 ± 0.36 and a ΔCt of 10.35 ± 0.70 for *KRT19* and *CEA* markers, respectively. For mesenchymal markers, a ΔCt of 8.21 ± 0.37 for *COL11A1* and ΔCt of 8.44 ± 0.37 for *COL1A2* were found (Table 2). Regarding CP expression analysis, epithelial marker ΔCt amplification values were 3.14 ± 0.30 for *KRT19* and 5.06 ± 0.75 for *CEA*. *COL11A1* showed a ΔCt value of 3.01 ± 0.26, and *COL1A2*, 3.27 ± 0.23 (Table 3). *COL1A2* expression was only analyzed in eleven cancer individuals due to the lack of sample and the impossibility of reproducing replicates in the remaining oncological patients.

**Figure 4:**
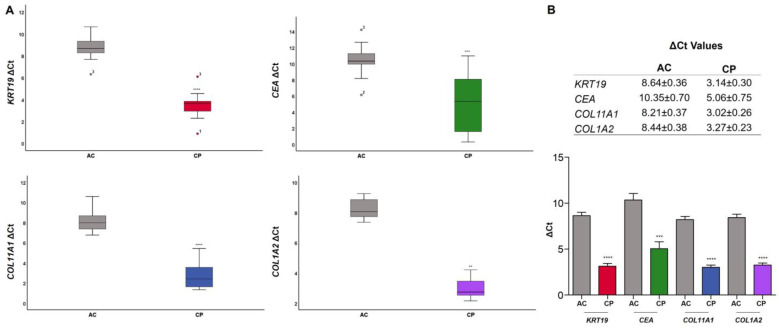
ΔCt values of epithelial and mesenchymal biomarkers (*KRT19*, *CEA*, *COL11A1*, and *COL1A2*) in AC and CP individuals. (**A**) Box plot representation showing the distribution of ΔCt values of each gene studied. The median ΔCt value is represented as a black line within the box plot, and it divides the ΔCt values into lower and upper quartile ranges. The whiskers represent the upper and lower data range in AC and CP samples. Data showed significant differences in ΔCt values between two cohorts of patients. (**B**) Comparison of ΔCt values of *KRT19*, *CEA*, *COL11A1*, and *COL1A2* in AC and CP samples. *t*-Tests showed significant different expressions between groups in all biomarkers (*KRT19*, *p*-value < 0.0001; *CEA*, *p*-value = 0.0001; *COL11A1*, *p*-value < 0.0001; *COL1A2*, *p*-value < 0.0001). Data are expressed as mean ± SE. Asterisks denote significant differences between groups AC and CP for each gene (**** *p*-value < 0.0001; *** *p*-value < 0.001; ** *p*-value < 0.01).

A box plot diagram was performed to visualize the distribution of results of ΔCt in AC and CP, and comparisons between both groups were made (Figure 4). The distribution of the data showed that the median ΔCt for *KRT19* in AC and CP was 8.66 (IQR: 1.21) and 3.66 (IQR: 1.24), respectively. The median ΔCt for *CEA* was 10.32 (IQR: 2.10; AC) and 5.32 (IQR: 6.92; PC). This last result indicated a great dispersion and variability of the data of the CP group for *CEA*. Stromal markers presented a median ΔCt for *COL11A1* of 7.97 (IQR: 1.54) in healthy donors and 2.39 (IQR: 2.03) for CP; and a median ΔCt for *COL1A2* of 8.29 (IQR: 1.64; AC) and 3.25 (IQR: 1.47; CP) (Figure 4A). Box plot diagrams also showed the presence of extreme data in ΔCt values in *KRT19* (AC and CP samples) and in *CEA* AC results. Grubbs and Dixon test analyses of the outliers were performed for these values and confirmed that these data were furthest from the rest but were not significant outliers (*p*-value > 0.05; IC: 95%).

Figure 4A,B also showed that differences in ΔCt values between AC and CP groups were significative in all genes studied. *t*-Tests yielded a *p*-value < 0.0001 for *KRT19*, *p*-value = 0.0001 for *CEA*, *p*-value < 0.0001 for *COL11A1*, and a *p*-value < 0.0001 for *COL1A2*.

**Figure 5:**
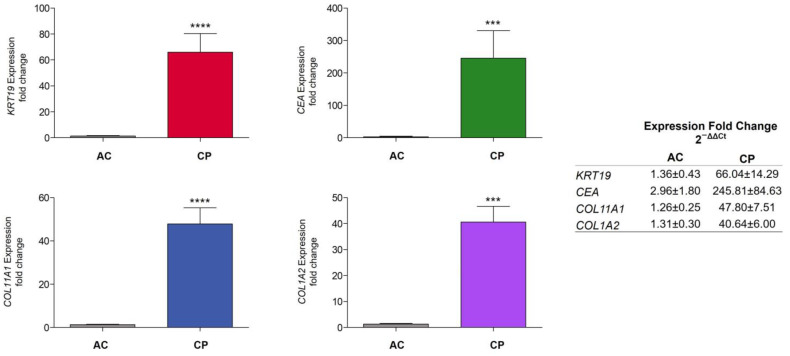
Relative fold expression of epithelial and mesenchymal biomarkers (*KRT19*, *CEA*, *COL11A1*, and *COL1A2*) between AC and CP cohorts of patients. Comparison of RNA relative expression of *KRT19*, *CEA*, *COL11A1*, and *COL1A2* in AC and CP samples by the 2^−ΔΔCt^ method. Data showed higher expression levels of *KRT19*, *CEA*, *COL11A1*, and *COL1A2* in CP individuals. Mann–Whitney U tests showed that these differences were significant between groups in all biomarkers (*KRT19*, *p*-value < 0.0001; *CEA*, *p*-value = 0.0003; *COL11A1*, *p*-value < 0.0001; *COL1A2*, *p*-value = 0.0001). Data are expressed as mean ± SE. Asterisks denote significant differences between groups AC and CP for each gene (**** *p*-value < 0.0001; *** *p*-value < 0.001.

The relative gene expression level was evaluated using a modified comparative Ct method, (2^−ΔΔCt^), as described previously by Pfaffl [53] for each biomarker. Figure 5 showed expression fold changes in AC and CP samples. *KRT19* showed an increase expression in CP (66.04 ± 14.29) in comparison to AC patients (1.36 ± 0.43). *CEA* levels also were highly incremented in cancer individuals (245.81 ± 84.63) related to healthy donors (2.96 ± 1.80). However, a high interindividual variability in expression was found for this gene in both AC and CP cohorts, even in oncological patients with the same type of cancer. Mesenchymal markers were as well highly elevated in CP group. AC patients showed values of 1.26 ± 0.25 (*COL11A1*) and 1.31 ± 0.30 (*COL1A2*), whereas CP expression for *COL11A1* was 47.80 ± 7.51-fold and 40.64 ± 6.00-fold for *COL1A2*. Both groups were found to be significantly different with a non-parametric test for all epithelial and mesenchymal biomarkers. Mann–Whitney U tests yielded a *p*-value < 0.0001 for *KRT19*, *p*-value = 0.0003 for *CEA*, *p*-value < 0.0001 for *COL11A1*, and *p*-value = 0.0001 for *COL1A2*.

**Figure 6:**
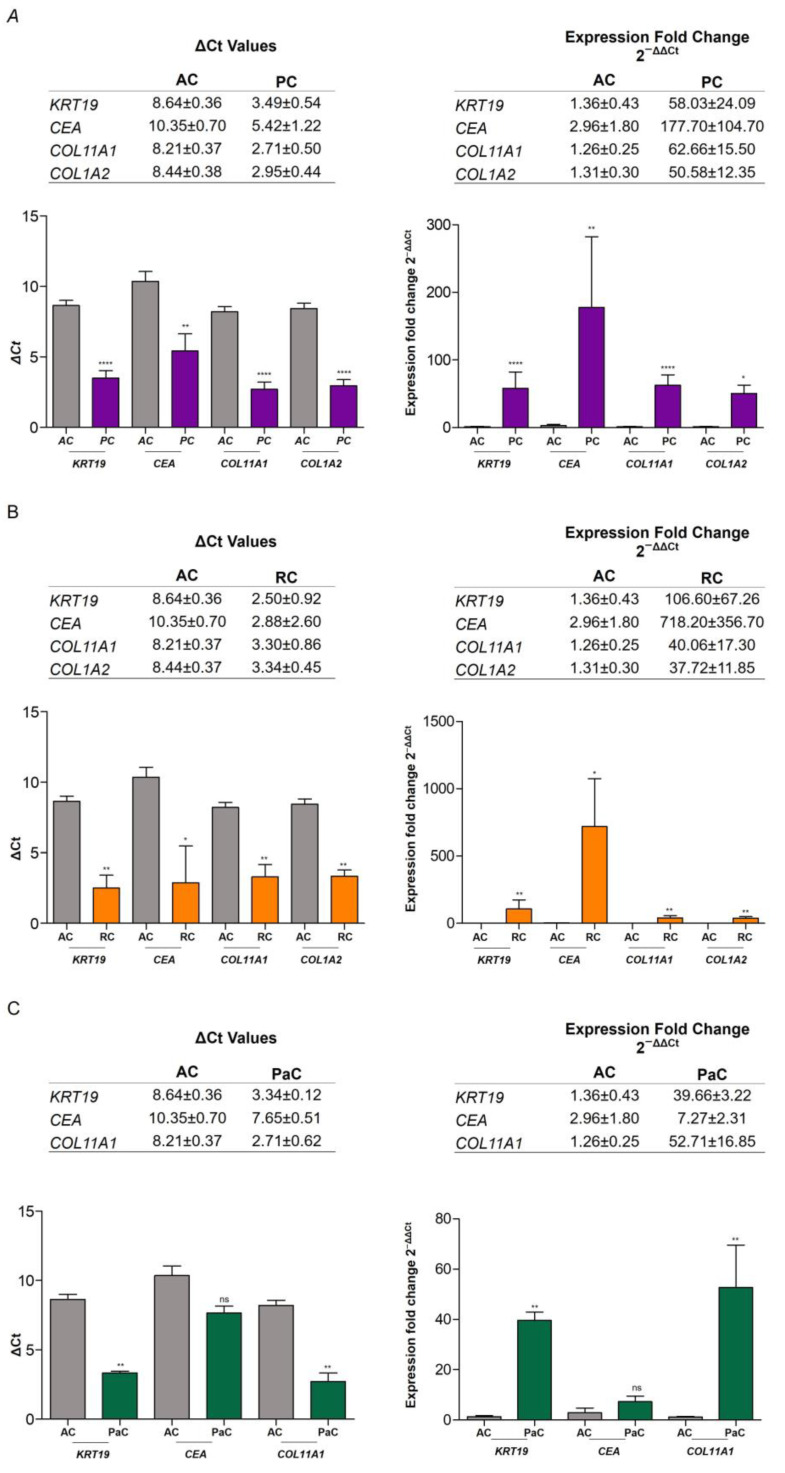
ΔCt values and relative fold expression of epithelial and mesenchymal biomarkers in AC and PC, RC and PaC individuals. (**A**) Comparison of ΔCt values and RNA relative expression by the 2^−ΔΔCt^ method of *KRT19*, *CEA*, *COL11A1*, and *COL1A2* in AC and PC samples. *t*-Tests showed significantly different ΔCt values between groups in all biomarkers (*KRT19*, *p*-value < 0.0001; *CEA*, *p*-value = 0.002; *COL11A1*, *p*-value < 0.0001; *COL1A2*, *p*-value < 0.0001). Mann–Whitney U tests also showed significant different expression between groups in all biomarkers (*KRT19*, *p*-value < 0.0001; *CEA*, *p*-value = 0.0087; *COL11A1*, *p*-value < 0.0001; *COL1A2*, *p*-value = 0.02). (**B**) Comparison of ΔCt values and RNA relative expression by the 2^−ΔΔCt^ method of *KRT19*, *CEA*, *COL11A1*, and *COL1A2* in AC and RC samples. Mann–Whitney U tests showed significantly different expression by ΔCt values and the 2^−ΔΔCt^ method between groups in all biomarkers (ΔCt: *KRT19*, *p*-value = 0.007; *CEA*, *p*-value = 0.022; *COL11A1*, *p*-value = 0.007; *COL1A2*, *p*-value = 0.007. Relative fold expression: *KRT19*, *p*-value = 0.007; *CEA*, *p*-value = 0.022; *COL11A1*, *p*-value = 0.007; *COL1A2*, *p*-value = 0.007). (**C**) Comparison of ΔCt values and RNA relative expression by the 2^−ΔΔCt^ method of *KRT19*, *CEA*, and *COL11A1* in AC and PaC samples. Mann–Whitney U tests showed significantly different expression by ΔCt values and the 2^−ΔΔCt^ method between groups in *KRT19* and *COL11A1* but not in *CEA*. (ΔCt: *KRT19*, *p*-value = 0.007; *CEA*, *p*-value = ns; *COL11A1*, *p*-value = 0.007. Relative fold expression: *KRT19*, *p*-value = 0.007; *CEA*, *p*-value = ns; *COL11A1*, *p*-value = 0.007). Data are expressed as mean ± SE. Asterisks denote significant differences between groups for each gene (**** *p*-value < 0.0001; ** *p*-value < 0.01; * *p*-value < 0.05; ns = not significant).

Regarding the gene expression results obtained with the 20 cancer patients in total, we separated the patients according to the type of tumor and analyzed them individually. Only the pooled data of patients with prostate (*n* = 8), renal (*n* = 3), and pancreatic cancer (*n* = 3) were discussed, as they were the only ones with a statistically significant number of cases. Data of *COL1A2* expression in pancreatic cancer patients were not analyzed due to the lack of samples and the impossibility of reproducing replicates. ΔCt values and RNA relative expression data are collected in Figure 6. In prostate cancer patients (PC), the results obtained were very similar to analyzed data with the total number of patients (Figure 6A). Both, ΔCt and 2^−ΔΔCt^ values showed a significant increase in all markers compared to the AC cohort. *KRT19* and *CEA* levels showed increased expression in PC (58.03 ± 24.09; 177.70 ± 104.70, respectively). The relative fold expression values of mesenchymal markers in the PC cohort were also higher (*COL11A1* = 62.66 ± 15.50; *COL1A2* = 50.58 ± 12.35). Concerning renal cancer patients (RC), we observed the same results (Figure 6B). The differences in expression in epithelial markers compared to healthy donors were even superior (*KRT19* = 106.60 ± 67.26; *COL1A2* = 718.20 ± 356.70), probably due to the small number of cases analyzed. In pancreatic cancer patients (PaC), *CEA* levels were found not significant (Figure 6C). *KRT19* (39.66 ± 3.22) and *COL11A1* (52.71 ± 16.85) showed higher expression levels in the PaC cohort compared to AC (*KRT19* = 1.36 ± 0.43; *COL11A1* = 1.26 ± 0.25). No data of *COL1A2* expression were available. In summary, analyzing the cases according to the type of tumor, we found similar expression levels to the complete CP cohort. However, further studies with a larger number of patients are necessary to be able to assess each type of tumor individually.

#### 2.4.2. Analysis of Specificity of cDNA Amplicons

**Figure 7:**
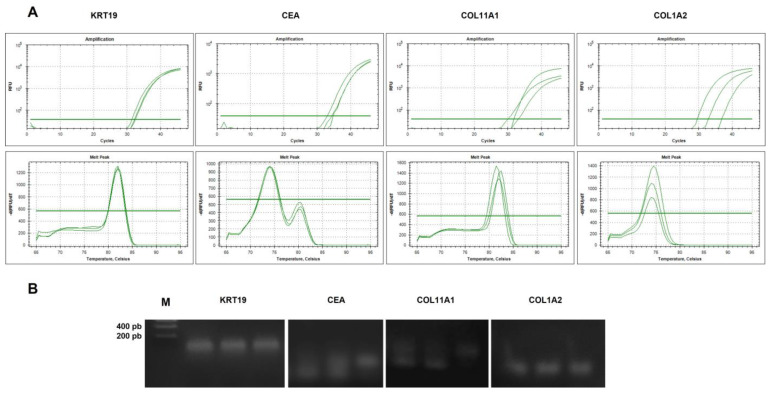
Analysis of specificity of PCR products. A. Amplification and melt curves of *KRT19*, *CEA*, *COL11A1*, and *COL1A2* in three cancer patients from a real time PCR assay. Sigmoidal curves in log scale were shown for both epithelial and mesenchymal biomarkers (upper zone). (**A**) Single peak of specific PCR amplicons was found for *KRT19*, *COL11A1*, and *COL1A2* samples (lower zone). The *CEA* curves showed two identified peaks at different temperatures, revealing the presence of primer dimers and/or artifacts. The dissociation temperature range of melt curves extended from 55 °C to 95 °C. (**B**) Agarose gel electrophoresis of epithelial and mesenchymal marker PCR products. *KRT19* and *COL1A2* amplicons showed a single specific band of less than 200 pb. *COL11A1* analysis revealed amplification products with a double band. All samples of *CEA* showed the presence of two bands of similar size.

We analyzed the specificity of the PCR products obtained for all biomarkers by analyzing the melt curves for each gene and summiting the amplified fragments to 2% agarose gel electrophoresis in order to verify amplicon size and discard the presence of dimers and other nonspecific products that could be interfering with the results obtained. Results are shown in Figure 7. Data representing three CP samples showing amplification sigmoidal curves in the log scale for both epithelial and mesenchymal biomarkers (Figure 7A, upper zone). Regarding melt curves (Figure 7A, lower zone), *KRT19*, *COL11A1*, and *COL1A2* curve analysis revealed a single peak of specific PCR products in all samples. This single peak was distributed in the same specific temperature range for each pair of primers. However, *CEA* curves showed two peaks at different temperatures. Specific products of real-time PCR had a higher melting temperature than nonspecific products (primer dimers or artifacts) and a smaller peak. Melt curve analysis was performed in all samples (AC and CP), and *CEA* results were similar in all runs. Others sets of *CEA* pairs of primers were proved, but no significant differences were found. The negative control for *CEA* also evidenced unspecific peaks in curve analysis.

Agarose gel electrophoresis displayed analogous results (Figure 7B). *KRT19* and *COL1A2* amplicons showed a very specific band without the presence of dimers or artifacts. Nevertheless, *COL11A1* and *CEA* showed two bands of similar size, more identified in *CEA* samples. All *CEA* amplicons analyzed by electrophoresis presented two amplified PCR products; however, in only a part of *COL11A*1 amplicons was a double band revealed. An analysis of *ACTB* PCR products was also performed by a study of melt curves and agarose gel electrophoresis showing specific amplicons for the control gene.

## 3. Discussion

Molecular diagnostics is already an integral part of practical medicine in oncological processes, but due to the complex and changing nature of the disease, robust and well-validated cancer biomarkers are increasingly needed [54]. World Health Organization defined a biomarker as “any substance, structure or process that can be measured in the body or its products and influence or predict the incidence of outcome or disease” [55,56]. Regarding cancer clinical practice, a cancer biomarker may measure the risk of developing cancer, cancer progression, or potential responses to therapy [57]. TD-EVs found in liquid biopsies have the unique potential to capture the dynamic landscape of the disease, and their physiological features make them potential vehicles for biomarkers in cancer [58]. In this sense, by analyzing different epithelial and mesenchymal markers extracted from plasmatic EVs, we found significant differences in gene expression between healthy donors and patients with active oncological disease. Our results at 10 AC and 20 CP showed that the isolated EVs by STEM and NTA contained a majority of exosome structures with also a considerable percentage of microvesicles. Surprisingly, no differences were found in concentration and size distribution between the two cohorts of patients [33,59]. However, this fact is not that unusual, since it depends on the type or physiology of the patient, and other studies have not found these differences between the two groups either [60,61]. Furthermore, several studies showed that age significantly influences the secretion of EVs [62,63]. Plasma concentrations of EVs may decrease with age, and this difference could be bigger according to the difference in age ranges. Our AC group presented a mean age of 40.30 years, while the mean age of the CP was 67.50 years. Therefore, the baseline characteristics of our two cohorts of patients could be decisive in this subject.

Analyzing the total extracted RNA from plasmatic EVs, we observed a composition of diverse specimens with a higher percentage of small RNAs than in cells. Low concentrations of total RNA were also yielded, but the amount was enough for performing replicable qRT-PCR analysis. Moreover, despite the low RIN values observed, probably because it is not an adequate method to measure the total RNA of EVs, we obtained specific amplicons of the target genes in our CP cohort. Although RNA is more vulnerable and less stable than DNA, it is well preserved in various body fluids when secreted as part of EVs. Furthermore, RNA secretion is a physiological process; it is secreted from active living cells rather than apoptotic/necrotic cells, which gives very relevant information on the origin and may be significant in some types of cancer that secreted a low abundance of tumor-derived plasma DNA [54]. RNA-based assays with validated results have been under development for years [64,65] and can provide a complete overview of the expression profiles that may be occurring at a given point in time in the pathology. Therefore, despite the fact that low concentration levels may be a limiting factor, the analysis of RNA extracted from TD-EVs could be a correct approach to develop a diagnostic tool.

Quantitative PCR results showed significant differences in both epithelial and mesenchymal expression between healthy and cancer patients. *KRT19*, *CEA*, *COL1A2*, and *COL11A1* expression levels were much higher in cancer patients (*KRT19* = 66.04 ± 14.29-fold; *CEA* = 245.81 ± 84.63-fold; *COL11A1* = 47.80 ± 7.51-fold; *COL1A2* = 40.64 ± 6.00-fold). Marker expression in healthy controls was almost residual in all cases. Analyzing data according to the type of tumor, we observed similar results in prostate, renal, and pancreatic cancer. Our data revealed that *CK19*, *COL11A1*, and *COL1A2* could be potential tumor biomarkers in plasmatic EVs, since results both at the level of expression as well as specificity and reproducibility are robust and promising. However, RNA expression findings obtained for *CEA*, both in healthy donors and cancer patients (in complete cohort and individually by tumor type), were not solid and reliable. This fact could explain the high variability found in the results, so the *CEA* biomarker, in those conditions, does not appear to be a suitable marker for our diagnosis system.

A cancer biomarker test should be an assay easily and reproducibly performed and must meet several main requirements. High specificity and sensibility are needed, allowing for the accurate discrimination between cancer and other pathological or physiological processes. Moreover, ideally, proportionality must also be taken into account, correlating with diverse features of malignancy as a stage or prognosis [65,66]. In our study, all the patients were in an advanced stage of the disease; however, our preliminary results encourage us to carry out further studies with patients with earlier stage disease in order to validate our data. Multicancer screening based on blood analysis may have the potential to be applied to early detection and could also address some limitations of current screening methods. There are currently very few multicancer tests on the market and with administration approval. CancerSEEK was one of the first multicancer early detection tests being reported. It is a blood test that can detect eight different types of cancer by the detection of cfDNA and eight protein biomarkers that are released by tumors [67]. Sensitivity of the test ranged from 69 to 98%, and specificity was greater than 99%. However, is not yet available to patients and it is awaiting FDA approval. The Galleri Test [68] detects abnormalities in the methylation patterns of cfDNA through next-generation sequencing. The test detects over 50 types of cancer and can predict the organ of origin of the cancer signal. Specificity is 99.5%, and overall sensitivity is 51.5%. It is FDA approved but is still only commercialized in America. The Panseer test [69] detects DNA methylation patterns linked to gene silencing that may contribute to cancer development. The test could detect five common types of cancer in 88% of post-diagnosis patients with a specificity of 96% and could detect cancer in 95% of asymptomatic individuals who were later diagnosed. One of the limitations of the test is that it does not detect the tissue of origin, and it is for research use only. The development of a diagnostic tool is a long process that usually takes place over different phases, namely, an initial discovery and assay development, the assessment of clinical validity, and finally market approval. As we have seen above, there are very few tests available for healthcare assistance, so the search for new fast and non-invasive multicancer early detection tools seems crucial nowadays. The three markers in which we obtained acceptable results (*KRT19*, *COL1A2*, and *COL11A1*), if used in combination, could increase the discriminatory potential that each one showed separately. Validation studies with a larger number of patients in early stages of the disease are needed to ensure statistical robustness of the assay. Moreover, samples should reflect the biological variability of the targeted population, intending to differentiate subjects according to the different types of tumors.

In conclusion, the role of plasmatic EVs in the progression of oncological diseases, as well as their therapeutic potential [70], together with the specificity of neoplastic transformation indicators such as *KRT19* or diverse CAFs markers, leads us to consider that the development of a non-invasive cancer diagnosis system using a combination of epithelial and mesenchymal markers could be a promising approach in the diagnosis of several neoplastic processes.

## 4. Materials and Methods

### 4.1. Patients and Human Samples

This research was conducted as a single-center retrospective study between February 2020 and March 2020. Samples were collected and analyzed in the Pathological Anatomy service at the University Hospital Complex of A Coruña. The Pathological Anatomy laboratory is UNE-EN ISO 9001-2015 certified. Clinical data such as gender, age, tumor histology, and disease stage were obtained from the medical records. Patients belonged to a study approved by the Clinical Research Ethics Committee (approval registration number 2020/010), and it was conducted in compliance with the Declaration of Helsinki. Written informed consent custody and remnant sample storage was managed by the Biobank of A Coruña.

A total of 30 plasma samples belonging to 30 patients was included in this study. Ten healthy AC and 20 CP that presented advanced clinical stage (III-IV) at the moment of the analysis underwent blood extraction for healthcare causes. Remnant samples were used for the study.

### 4.2. Blood Sample Collection, Extracellular Vesicle Isolation, and RNA Purification

Peripheral whole blood was collected from each subject in a 10 mL EDTA-K2 tube and processed after centrifugation within 4 h to avoid contamination with genomic DNA released from lysed blood cells. Samples were centrifuged at 2000× *g* for 20 min to collect 2 to 4 mL of plasma The plasma obtained was passed through a 0.8 μm filter and stored at −80 °C. Processing of plasma samples and RNA isolation was carried out using the commercial ExoRNeasy Maxi Kit (QIAGEN, Hilden, Germany), and the manufacturer’s protocols were followed. Briefly, a membrane-based affinity binding step to isolate EVs from filtered plasma was used. Subsequently, a phenol/guanidine-based combined lysis and elution step recovered vesicular RNA from the spin columns. Purification of total RNA was performed by a silica-membrane-based column system. Total RNA was eluted in 14 μL of RNase-free water. Purified RNA from each sample was assayed qualitatively and quantitatively using the Agilent RNA 6000 Pico Kit (Agilent Technologies, Santa Clara, CA, USA) on an Agilent 2100 Bioanalyzer (see protocol hereafter).

### 4.3. Extracellular Vesicle Characterization by Scanning Transmission Electron Microscopy

To characterize ultrastructural morphology of plasmatic EVs obtained from patients, STEM was performed. EVs were isolated as describe above. After collection, EVs were resuspended in 500 μL of XE buffer (QIAGEN, Hilden, Germany), and samples were then adsorbed onto 300-mesh carbon-coated copper grids for 1 min in a humidified chamber at room temperature. Grids with adhered EV were examined with a Zeiss Gemini SEM 500 microscope (Carl Zeiss Microscopy GmbH, Jena, Germany) equipped with a STEM detector at 20–30 kV.

### 4.4. Extracellular Vesicle Characterization by Nanoparticle Tracking Analysis

The size distribution and concentration of plasmatic EVs was determined using a Malvern NanoSight NS300 Analyzer (Malvern Panalytical Ltd., Malvern, UK) with specific parameters according to the manufacturer’s protocols. EVs were isolated using the ExoRNeasy Maxi Kit (QIAGEN, Hilden, Germany) following manufacturer’s instructions and resuspended in 500 μL of XE buffer (QIAGEN, Hilden, Germany). Captures and analysis were achieved by using the built-in NanoSight Software NTA3.3.301 (Malvern Panalytical Ltd., Malvern, UK). The detection threshold for nanoparticles was fixed at 8 for all tests. Samples were diluted in PBS to a final volume of 1 mL. For each measurement, five consecutive 60 s videos were recorded at 25 °C, using a continuous syringe pump at an infusion rate of 40 units. Particles (EVs) were detected using a 488 nm laser (blue) and a scientific CMOS camera.

### 4.5. Bioanalyzer Analysis of Total Purified RNA

Quality, integrity, and the size distribution pattern of total RNA was analyzed using chip-based capillary electrophoresis Agilent 2100 Bioanalyzer using the RNA 6000 Pico Chip (Agilent Technologies, Santa Clara, CA, USA), according to manufacturer’s protocol. The RNA 600 Pico Chip assay is designed for analysis of RNA fragments, and each chip contains an interconnected set of microchannels that is used for separation of nucleic acid fragments based on their size. Quality and quantity measures were collected from the generated Bioanalyzer result reports and after evaluation of the reference ladder. The total RNA concentration in the sample had to be between 200 and 5000 pg/μL.

### 4.6. Reverse Transcription and Real-Time PCR (qRT-PCR)

#### 4.6.1. Reverse Transcription

RNA was quantified by an Agilent 2100 Bioanalyzer, and total RNA samples were reverse transcribed into cDNA according to the QuantiNova Reverse Transcription kit (QIAGEN, Hilden, Germany) protocol. The synthesis of cDNA was carried out at a thermal cycler following the manufacturer’s instructions: 2 min at 45 °C for gDNA elimination reaction, 3 min at 25 °C for annealing step, 10 min at 45 °C for reverse transcription step, and 5 min 85 °C to inactivate the reverse transcriptase.

#### 4.6.2. qRT-PCR

Quantitative RT-PCR was performed in a CFX96 C1000 Thermal Cycler (Bio-Rad Laboratories, Hercules, CA, USA). cDNA expression was assessed using a QuantiNova SYBR Green PCR kit (QIAGEN, Hilden, Germany). PCR conditions were set according to the supplier. Briefly, initial activation at 95 °C for 2 min and 45 cycles of 95 °C for 5 s, 60 °C for 10 s. Analysis of the melting curves: increase of 0.5 °C from 55 °C to 95 °C. PCRs were performed in 20 μL reaction volumes containing 6 μL H_2_O, 10 μL QuantiNova SYBR Green PCR, 1 μL of each primer (20 µM forward/reverse primers (TIB Molbiol, Berlin, Germany), final concentration: 1 µM), and 2 μL cDNA template. Forward and reverse primers are listed in Table 4.

Relative gene expression was calculated using a modified comparative threshold cycle (Ct) method, (2^−ΔΔCt^), as described previously by Pfaffl [53]. The method is a simple formula used in order to calculate the relative fold gene expression. Fold gene expression = 2^−ΔΔCt^, where ∆Ct = average of Ct (gene of interest) − average of Ct (housekeeping gene); and ∆∆Ct = average of ∆Ct (group of interest) − average of ∆Ct (control group). Two replicates of each sample were analyzed for each gene. For housekeeping gene (*ACTB*), a Ct ≤ 28 was considered positive. *ACTB* Ct values ≥ 29 were considered negative and results were considered invalid. For epithelial and mesenchymal markers, a Ct value ≤ 39 with a sigmoidal curve was accepted as positive. After amplification, a representative sample from each set of amplicons was analyzed by agarose electrophoresis to confirm their specificity. Sixteen microliters of PCR products were separated by electrophoresis on a 2% agarose gel.

### 4.7. Statistical Analysis

Statistical analyses were performed using the IBM SPSS^®^ Statistics v27 program. Descriptive statistics were used for characterizing the clinical and pathological data of the patients in the study. A Shapiro–Wilk normality test was performed for each data set. A Grubbs and Dixon test was carried out to detect atypical data (outliers). Box plot diagrams were performed for study the distribution of ΔCt values between AC and CP groups. *t*-Tests were performed for comparisons between two groups when the statistical data followed a normal distribution (comparison of ΔCt values between AC and CP groups for epithelial and mesenchymal biomarkers). The nonparametric Mann–Whitney U test was used for comparisons of relative fold expression (2^−ΔΔCt^ values) among the two cohort of patients for each gene. ΔCt and 2^−ΔΔCt^ values were represented as mean ± SEM. Statistical significance was determined at α-limit = 5%.

## 5. Patents

B.O.A., L.S.E.-P., M.O.A., S.D.H., L.S. and Á.C. filed a patent application that details the potential role of plasmatic TD-EV RNA of KRT19 and COL1A2 as a non-invasive diagnostic tool in the diagnosis of several neoplastic processes.

## Figures and Tables

**Table 1:** Baseline characteristics of the patients.

**Clinical and Pathological Features of Patients Included in This Study (*n* = 30)**			
**Clinic pathological features**		**AC (*n* = 10)**	**CP (*n* = 20)**
Age median (range)		40.30 (25–55)	67.50 (41–91)
Gender			
Female		7 (70.00%)	3 (15.00%)
Male		3 (30.00%)	17 (85.00%)
**Clinical and Pathological Features of Patients with Tumoral Process (*n* = 20)**			
**Prostate cancer (PC) (*n* = 8)**		**Renal cancer (RC) (*n* = 3)**	
Age mean (range)	73.25 (64–91)	Age mean (range)	63.67 (63–65)
Histological type		Histological type	
Adenocarcinoma	8	CCRC	3
Clinical stage		Clinical stage	
Tis/I/II	1	Tis/I/II	0
III/IV	7	III/IV	3
**Pancreatic Cancer (PaC) (*n* = 3)**		**Melanoma (Me) (*n* = 2)**	
Age mean (range)	50.33 (41–65)	Age	57–83
Histological type		Histological type	
Neuroendocrine carcinoma	3	Melanoma	2
Clinical stage		Clinical stage	
Tis/I/II	0	Tis/I/II	1
III/IV	3	III/IV	1
**Others (*n* = 4)**			
	Age	Histological Type	Clinical stage
Bladder carcinoma	73	Urotelial carcinoma	IV
Colorectal carcinoma	63	Adenocarcinoma/neuroendocrine carcinoma	I/IV
Ovarian cancer	70	Papillarly serous carcinoma	III
Lung cancer	76	Small-cell carcinoma	IV

**Table 2:** ΔCt amplification values for *KRT19*, *CEA*, *COL11A1*, and *COL1A2* genes corresponding to asymptomatic controls (AC). ΔCt indicates the difference between the mean of two replicates of the Ct of the target gene and the mean of the Ct of the reference control gene (*ACTB*).

Patient	Sex	RNA Expression			
		ΔCt *KRT19*	ΔCt *CEA*	ΔCt *COL11A1*	ΔCt *COL1A2*
AC1	Female	8.27	8.16	10.59	9.25
AC2	Female	8.58	6.12	9.5	7.36
AC3	Female	6.31	14.21	8.27	8.85
AC4	Male	7.68	10.32	6.76	9.35
AC5	Female	8.67	10.56	7.54	10.81
AC6	Male	9.35	9.95	8.67	7.73
AC7	Male	9.32	11.25	7.36	6.64
AC8	Female	8.65	10.32	8.45	8.27
AC9	Female	10.65	9.99	7.25	7.79
AC10	Female	8.95	12.65	7.68	8.32
	Mean	8.64	10.35	8.21	8.44
	SEM	0.36	0.70	0.37	0.37

**Table 3:** ΔCt amplification values of *KRT19*, *CEA*, *COL11A1*, and *COL1A2* genes corresponding to cancer patients (CP). ΔCt indicates the difference between the mean of two replicates of the Ct of the target gene and the mean of the Ct of the reference control gene (*ACTB*).

Patient	Stage	Sex	RNA Expression			
			ΔCt *KRT19*	ΔCt *CEA*	ΔCt *COL11A1*	ΔCt *COL1A2*
Prostate cancer	IV	Male	0.88	5.41	1.33	2.59
Prostate cancer	IV	Male	3.84	6.7	2.51	2.87
Prostate cancer	IV	Male	6.09	10.97	3.66	4.2
Prostate cancer	IV	Male	2.29	0.68	2.28	
Prostate cancer	IV	Male	3.61	3.92	5.42	
Prostate cancer	IIB	Male	3.71	1.57	1.62	2.15
Prostate cancer	IV	Male	2.94	5.24	1.32	
Prostate cancer	IV	Male	4.55	8.87	3.55	
Renal cancer	IV	Male	3.81	8.07	2.81	3.45
Renal cancer	IV	Male	2.97	0.28	2.11	2.52
Renal cancer	III	Male	0.73	0.28	4.98	4.06
Pancreatic cancer	IV	Male	3.15	6.84	2.11	
Pancreatic cancer	IV	Male	3.56	8.58	3.96	
Pancreatic cancer	IV	Male	3.31	7.54	2.07	
Melanoma	0	Male	1.51	6	2.99	3.25
Melanoma	IV	Female	3.02	9.42	4.58	2.78
Bladder carcinoma	IV	Female	3.18	1.93	2.98	3.61
Colorectal carcinoma	IV	Male	5.34	4.61	3.65	4.44
Ovarian cancer	III	Female	2.1	3.53	3.25	
Lung cancer	IV	Male	2.29	0.73	3.1	
		Mean	3.14	5.06	3.01	3.27
		SEM	0.30	0.75	0.26	0.23

**Table 4:** Primers used in qRT-PCR.

Primer Name	Forward Primers	Reverse Primers
*ACTB* (Housekeeping)	5′AGCCTCGCCTTTGCCGA 3′	5′CTGGTGCCTGGGGCG 3′
*KRT19*	5′CAGCCACTACTACACGACCATC 3′	5′CAAACTTGGTTCGGAAGTCATC 3′
*CEA*	5′AATGGGATACCGCAGCAAC 3′	5′GAGAGACCAGGAGAAGTTCCAGAT 3′
*COL11A1*	5′AATGGAGCTGATGGACCACA 3′	5’TCCTTTGGGACCGCCTAC 3′
*COL1A2*	5′TCAAACTGGCTGCCAGCAT 3′	5’CAAGAAACACGTCTGGCTAGG 3′

## Data Availability

The data presented in this study are available in the article. Any other related information or document not present in this study are available upon request.
